# Standards of Conducts for Biostatisticians and Stem Cell Researchers: A Call for Self-formulated Aspirational Ethics Over Built-in Prohibitive Ethics

**DOI:** 10.1007/s11948-022-00366-5

**Published:** 2022-03-17

**Authors:** Keiko Sato, Mika Suzuki

**Affiliations:** 1 Department of Health Informatics, Kyoto University School of Public Health, Yoshida Konoecho, Sakyo-ku, Kyoto, 606-8501 Japan; 2 Uehiro Research Division for iPS Cell Ethics, Center for iPS Cell Research and Application, Kyoto University, Kyoto, Japan

**Keywords:** Standard of conduct, Aspirational ethics, Biostatistician, Stem cell researcher, Virtue ethics, Research governance

## Abstract

We proposed the Standards of Conducts to provide a general framework that will serve as the basis for guiding each biostatistician and stem cell researcher to formulate their personal standards, rather than as rules with which they are required to comply. Given the responsibility and characteristics of their work, they are expected to maintain independence and work autonomously as professionals. Each of the Standards of Conducts comprises a preamble, mission and values to uphold, Standards of Conducts (10 items), and background. When one internalizes “self-formulated” standards, to make excuses for oneself would be akin to a self-betrayal; responsible actions can be anticipated. If one begins and continues to consider “who I am and what do I work for,” this will become their inner energy, and a source of motivation and pride to inspire oneself. In addition, this aspirational style might help citizens to recognize the autonomous stance of the professional body and that they share the same values.

## Introduction

A code of ethics is the culmination of standards for a professional group to act appropriately; it guides individual members of the profession through the decision-making process when confronted with ethical issues (Davis, 1991). It also serves as a tool for the public to know the foundation upon which standards have been built for professionals to abide by. For this reason, an academic group develops a code of ethics with the aim to urge its members to follow rules that prohibit wrongdoing and prevent undesirable consequences. Although such codes consist mainly of prohibitory items, these are typically insufficient when facing new dilemmas which have never been encountered in practice.

Technologies advance rapidly in biomedical research, such as human genome editing, and changes are relatively large-scale (Morange, 2017). Thus, there is a trend to impose strict governance requirements and oversight rather than promote professionalism among specialists (Collins & Gottlieb, 2018). However, a strict oversight system may not necessarily ensure that research is conducted appropriately. This may lead researchers to believe that following the rules is an end in itself, or that doing things not prohibited by the rules is acceptable. Professionals are guided by their own curiosity and must maintain legitimacy by claiming academic freedom, self-direction, and self-regulation (Jones, 2007). To carry out good research, it is not sufficient to follow the rules. Rather, intellectual and moral excellence is necessary (Hursthouse, 2014). Researchers are required to be thoughtful about who they are and what they aspire to be, and to act responsibly. When faced with a dilemma, they must be able to make judgements consistent with their own value system and moral compass. This requires knowing what an appropriate action is, acting in accordance with what one considers appropriate, having the disposition to act in accordance with the right middle, and being prepared to act accordingly in each situation on a regular basis (Aristotle, 2014). Moreover, these judgements are not to be imposed on one by another but must come from within each researcher. Accordingly, we have developed Standards of Conduct (SOC) in order to provide a framework to encourage each researcher to formulate their personal standards based on universal principles in order to ensure good practice and advance the interests of society.

In this article, we will first explain the limitations of heteronomous rules and the need for autonomous SOC. This will be followed by the background and process by which we developed SOCs for biostatisticians and stem cell researchers (SCRs). We then present the content and characteristics of both SOCs and provide an outline for a seminar designed to encourage biostatisticians to formulate their personal standards and foster professionalism as a practical example. We conclude the discussion by emphasizing the importance of “self-formulated” aspirational standards.

## Limitations of Heteronomous Rules

Following multiple scandals involving STAP cells and clinical trials of antihypertensive drugs, Japan’s ministries have taken measures against recurrence by implementing stricter heteronomous rules, through either the creation or improvement of laws and regulations (Science Council of Japan, 2013; Tashiro, 2018). While enforcing heteronomous rules is necessary to prevent recurrence, if appropriate research activities are to be promoted, professionals must do more than simply follow the rules established by someone else. Rather, they must act as professionals to personally uphold universal principles, contemplating and judging what is appropriate by themselves (Oakley & Cocking, 2006). Through collaborative work with biostatisticians and SCRs, we bioethicists came to believe that this would only become possible if they are fully and personally committed to “self-formulated” standards that serve as a foundation for judgment.

The work of biostatisticians and SCRs greatly impacts human life. As their practices uniquely demand advanced knowledge and skills, it is difficult for those outside these fields to understand the content or judge the appropriateness of their work. It is also essential for the work of biostatisticians and SCRs that citizens and patients feel secure about providing necessary data and biological samples, participate in clinical trials, and trust the results obtained (Sato et al., 2014). In the same way that “professionalism” is fundamental to medical and legal practices, these researchers must act responsibly, upholding “statistician/researcher-ship” and maintaining high ethical standards and autonomy, fully cognizant of the public nature of their practice (American Statistical Association, 2018).

Many biostatisticians belong to companies and research institutes. Thus, they may be subject to tangible and intangible pressures to protect the interests of their organizations when these organizations unfairly pursue their own interests (Sato et al., 2014). If biostatisticians prioritized the interests of their organizations, they would lose the trust of society. Furthermore, they may be put in difficult positions or find themselves in conflict with organizational interests if they try to fulfill their legitimate duties (Hurwitz & Gardenier, 2012). On the other hand, SCRs face various issues ranging from bioethical issues to emotional and religious issues, many of which are related to their privilege to use embryos to derive human ES cells, or to derive germ cells from human pluripotent stem cells (Lo & Parham, 2009). As biostatisticians and SCRs work behind closed doors, out of sight from public scrutiny, they are highly vulnerable to losing the trust of society if their behaviors deviate from social norms (Hyun et al., 2008; Jones, 2007). As such, they must judge for themselves what is acceptable research and clinical application, both socially and morally.

In Japan, bioethical issues, including research misconduct, are commonly dealt with in accordance with ethical guidelines set forth by relevant ministries or academic societies, and with which researchers are expected to comply. For instance, The Biometric Society of Japan has developed ethical standards (The Biometric Society of Japan, 2008). With regard to research using embryos, the Ministry of Education, Culture, Sports, Science and Technology formulated guidelines for research that establishes or uses human ES cell lines (The Ministry of Education, Culture, Sports, Science and Technology and The Ministry of Health, Labour and Welfare, 2019), and the Japan Society of Obstetrics and Gynecology issued “Views regarding research that involves use of human sperm, eggs, and embryos” (Japan Society of Obsterics & Gynecology, 2013). International ethical standards include the Declaration on Professional Ethics by the International Statistical Institute (ISI) (International Statistical Institute, 2010) and Guidelines for Stem Cell Research and Clinical Translation by the International Society for Stem Cell Research (ISSCR) (International Society for Stem Cell Research, 2016).

Many of these guidelines either take the form of prohibitive ethical codes with “dos” and “don’ts” or employ a heteronomous system in which normative guidelines are created and publicized by authorities such as ministries and academic societies, and then followed by researchers and members of academic societies. However, biostatisticians and SCRs require a high degree of professional responsibility, and thus the validity of their practices must be self-assessed. Moreover, given the fast pace of knowledge and technological innovations, as well as the daily emergence of new issues, no standards can be effective unless researchers themselves take the initiative to act consciously when fulfilling their roles and responsibilities (American Statistical Association, 2018). At the same time, biostatisticians and SCRs are expected by society to act proactively. They are expected “to conduct good research and good work” with active—not passive—attitudes rather than “to act without violating rules” (Harris et al., 2019).

## Need for Aspirational Standards of Conduct

We held a discussion among biostatisticians and SCRs to determine the ideal profile of someone in their profession (i.e., a professional). The following was established: they are those who shall respond to social expectations through activities that contribute to the enhancement of people’s well-being, while also acting with confidence and pride (Harris, 2008; Schinzinger & Martin, 2000), striving toward the realization of an environment in which the health of their existence can be maintained. We then established that, in order for individuals to become self-sufficient as professionals, they must formulate and hold personal standards within themselves.

To this end, we recognized the need to formulate a set of standards which would help professional bodies (i.e., academic societies) function in an autonomous fashion. To ensure that biostatisticians and SCRs are protected while fulfilling their roles, their involvement and roles must be clarified for their employers and collaborators. Accordingly, we established SOC for biostatisticians (SOC-B) and SOC for SCRs (SOC-SCR), which present values and universal principles that serve as the foundation for taking personal responsibility..

## Process and History Behind Development of the SOCs

The background leading to the development of SOC-B and SOC-SCR, including main comments by experts, are provided in Table 1. Regarding SOC-B, one of the authors (KS) was asked to join a taskforce for developing a “Code of Ethics” for biostatisticians. KS, an ethics consultant for clinical research, has experience in planning and managing clinical studies with biostatisticians who serve on the board of the Japanese Federation of Statistical Science Associations. SOC-B was drafted by a working group consisting of KS, five statisticians nominated by the Japan Statistical Society and the Japanese Society of Applied Statistics, and two statisticians from the ethics committee of the Institute of Statistical Mathematics, following discussions that began in 2007 in the taskforce and working group, and in response to the intention expressed by the chairman of the Biometric Society of Japan to revise the “Code of Ethics” in 2012. In 2013, following examination by the board of directors and councilors of the Biometric Society of Japan, SOC-B was adopted and published (The Biometric Society of Japan, 2013).

**Table 1:** Process of development of SOC for biostatisticians (SOC-B) and SOC for stem cell researchers (SOC-SCR)

	SOC-B	SOC-SCR
Timeline (year)	2007: A taskforce was formed by the chairman of the Japanese Federation of Statistical Science Associations, a biostatistician, and KS 2012: The chairman of the Biometric Society of Japan asked to develop Code of Ethics and organized a working group 2013: The working group drafted SOC-B 2013: The board of directors of the Biometric Society of Japan examined, adopted, and published SOC-B	2012: KS, a member of the ES cell line establishment project, proposed developing SOC-SCR based on lessons learned from the experience of developing SOC-B and organized a working group 2013: The working group drafted SOC-SCR 2014: The board of directors of the Japanese Society for Regenerative Medicine approved and published SOC-SCR following a review by the Society’s ethics committee
Working group members	• KS: Bioethicist • Members A, B: Biostatisticians who are members of the Japan Statistical Society • Members C, D, E: Biostatisticians who are members of the Biometric Society of Japan • Members F, G: Biostatisticians who are members of the ethics committee of the Institute of Statistical Mathematics	• KS, MS: Bioethicists who are members of the ES cell line establishment project and of the Japanese Society for Regenerative Medicine • Members H, I: Stem cell researchers of the ES cell line establishment project who are members of the Japanese Society for Regenerative Medicine • Member J: Research assistant of the ES cell line establishment project
Expert comments and views	<Previous Experience > • Inappropriate statistical methods have been used in the evaluation of new drugs because those in charge of statistical work lacked knowledge • The opinions of biostatisticians working in a pharmaceutical company have often been ignored due to their low status <Cultural and traditional background> • Japanese organizations have a seniority-based hierarchy, making it difficult for people to express their opinions openly and especially difficult to point out mistakes of their superiors • Even if one person says the right thing, it will be ignored, so the company needs to create a culture of ethical behavior < Information obtained from analysis > • Statistical work should be handled by qualified biostatisticians • If a statistician intends to commit fraud, it is difficult for non-statisticians to detect, so he or she must be virtuous • Biostatisticians need opportunities to acquire and refine knowledge and skills	<Previous Experience> • News reports that overemphasize ES cells as being ethically problematic (because they are created by destroying the blastocyst that will become a human being) or stir up excessive expectations for regenerative medicine as a dream treatment result in misunderstanding and hype among the public and politicians, thereby hindering the healthy progress of research • Misunderstanding and hype can also affect research funding allocation policies • There are problems with the method of formulating national guidelines for research using ES cells, which can reflect some committee members’ individual beliefs, resulting in overly strict regulations that hinder research <Information obtained from analysis> • Stem cell researchers need to inform the public about their mindset, including the proper handling of blastocysts and somatic cells • It is important for researchers to convey correct information to the public and tell the media to report news correctly • Researchers need to make sure they maintain appropriate relationships with government officials • A professional body should take the initiative to examine research issues, and practical discussions should take place rather than conceptual discussions
Use of SOC	• Members joining the Biometric Society of Japan are required to read SOC-B • In a seminar for biostatisticians to foster professionalism in universities and companies, participants (students and biostatisticians) are asked to formulate their personal standards based on SOC-B	• Members joining the Japanese Society for Regenerative Medicine are required to read SOC-SCR and sign a commitment form

With regard to SOC-SCR, when the Kyoto University Institute for Frontier Life and Medical Sciences started a project with the aim of deriving embryonic stem (ES) cell lines for possible clinical application from surplus blastocysts in 2012, KS and MS took charge of the informed consent process for potential blastocyst donors as bioethicists involved in this project. KS and MS proposed establishing SOC-SCR based on lessons learned from the experience of developing SOC-B and organized a working group consisting of KS, MS, two stem cell researchers, and a research assistant. SOC-SCR was drafted in 2013 and made public. Subsequently, a member of the working group and director of the Japanese Society for Regenerative Medicine approached the ethics committee of the society about adopting SOC-SCR, and after a review by the committee, SOC-SCR was approved and published by the board of directors in 2014 (Japanese Society for Regenerative Medicine, 2014).

## Important Points Considered During SOC Drafting

Both SOC-B and SOC-SCR comprise a Preamble, Mission and values to uphold, Standards of conduct (10 items), and Background, in accordance with the structure of the ISI standards (Table 2).

**Table 2:** Structure and summary of standards of conduct (SOC) for biostatisticians and stem cell researchers (SCRs)

Structure	SOC for biostatisticians	SOC for SCRs
Preamble	**Purpose** To offer a framework to encourage biostatisticians to formulate personal standards To present explicitly SOC to society, so that people can understand the responsibilities and activities of biostatisticians **Responsibility of biostatisticians** To have expert knowledge and skills To be aware of their social responsibility, recognize the public nature of their practice, and prioritize the interest of society To maintain independence and work autonomously **Necessity of professional group and SOC** To keep the quality of work and secure one’s position To earn trust and respect for the entire community of biostatisticians	**Purpose** To offer a framework to encourage SCRs to formulate personal standards To present explicitly SOC to society, so that people can understand the responsibilities and activities of SCRs **Responsibility of SCRs** To have expert knowledge and skills To be aware of their social responsibility, recognize the public nature of their practice, and prioritize the interest of society **Necessity of professional group and SOC** To earn trust and respect for the entire community of SCRs To consider about the extent to which stem cells can be utilized in research and medical care, and how a certain technology might be applied
Mission and Values	**Mission** To contribute to the promotion of people’s health, well-being, conservation of the environment, and the development of society and economy **Values** 1. Respect for human life and dignity To act with consideration for peoples’ lives, dignity, personality, and well-being, as well as the environment 2. Responsibility and skills To acquire expertise and skills To draw objective conclusions by designing a meaningful study design, collecting high quality data, and performing analyses using methods that fit the purpose 3. Act with honesty and integrity To work in a way that enables them to ensure the appropriateness of their work, accuracy of results, and validity of the science To build appropriate relationships with their employers and clients To describe their activities and outcomes with their grounds. To clarify used data, analysis results, and the method	**Mission** To contribute to people’s health, safety, and well-being, and to promote public health To encourage the healthy development of regenerative medicine research, as well as to practice and guarantee appropriate medical care **Values** 1. Respect for human cells, life, and dignity, and for the environment To handle blastocysts and somatic cells carefully as a treasure of society To ensure that clinical applications are done and findings are used towards the gain of society as a whole 2. Respect for cultural and social values and conduct of activities that respect the ideals/philosophies of regenerative medicine To refrain from activities that endanger these values, such as activities that go against public order or deviate largely from common sense To ensure the application is done within the framework of the ideals of regenerative medicine, the primary aim of which is to regenerate lost function in a biological tissue To ensure technologies are used in humans only after sufficient safety measures are taken To ensure an event that cannot be addressed using safety measures would happen only when the probability is such that responsibility will encompass only a limited number of individuals such as the patient, family, or medical personnel 3. Act with honesty and integrity To explain the objectives, methods, and significance of the findings of medical care and research to laypersons To evaluate and expound on risks and benefits
SOC	1. Exhibit professionalism 2. Carry out work properly 3. Clarify roles and responsibility to others 4. Publish and describe work and outcomes 5. Assess and prevent risks 6. Handle information appropriately 7. Comply with laws and guidelines 8. Respect human rights 9. Prevent misconduct 10. Prevent adverse effects of conflicts of interest	1. Exhibit professionalism 2. Carry out work properly 3. Evaluate risks and benefits appropriately 4. Select cell donors and subjects appropriately 5. Respect subject autonomy 6. Protect the welfare of donors and subjects 7. Maintain transparency and promote fluid communication with society 8. Comply with laws and guidelines 9. Prevent misconduct 10. Prevent adverse effects of conflicts of interest
Background Note	• Japan has experienced problems which included researchers interpreting the absence of significant difference as “equivalent” in the assessment of new drug efficacy and municipal officials falsifying data in national census surveys • It is essential for biostatisticians to earn the trust of society • Biostatisticians are expected to think and act proactively about problems • We formulated the basis for autonomous standards that urge biostatisticians to take an active stance • SOC can serve as a potential tool to protect biostatisticians when they are unduly influenced	• Regenerative medicine and pharmaceutical research using stem cells have progressed rapidly since human ES and iPS cell lines were successfully derived • Various problems now exist regarding the safety of using stem cells in humans • Risk/benefit assessment is difficult and researchers need to construct methods to evaluate efficacy or ensure quality control, which requires developing ways to foresee and address unprecedented situations • It is essential for SCRs to gain the trust of society for research to progress smoothly and for the public to feel at ease in donating cells or participating in clinical trials

SOC-B and SOC-SCR were drafted with reference to guidelines of relevant statistics societies/ISSCR and those of clinical research communities, such as the Belmont Report (The National Commission for the Protection of Human Subjects of Biomedical & Behavioral Research, 1979). Then, common values and universal principles to incorporate were extracted. Based on comments from experts and views obtained through discussions with biostatisticians and SCRs, issues unique to the environment of their activities, issues based on previous experiences, cultural, and traditional backgrounds, and information obtained from an analysis of the current situation were also taken into consideration, as detailed below.

### Standards of Conduct for Biostatisticians

Regarding the practice of biostatisticians, there has been a history of data fabrication in clinical trials or the use of incorrect statistical methods in non-inferiority trials for drug approval (Tsubaki, 2014). Main reasons include the lack of sufficient knowledge and skills among those in charge of drug licensing in the ministries and statisticians in charge of statistical work in pharmaceutical companies, and the lack of public understanding of the importance of biostatistics in the evaluation of new drugs. The opinions of statisticians are not always valued given their low position within a company, or because the hierarchical work environment makes it difficult for them to voice frank opinions to their supervisors due to the seniority-based corporate culture (Hofstede et al., 2010). Some opined that if a statistician intends to commit fraud, it would be difficult for non-statisticians to detect it, so he or she must be virtuous. Based on these opinions, we specified in SOC-B that people in charge of drug licensing and statistical work in companies should also need to acquire skills and establish themselves as professionals, and that pharmaceutical companies should cultivate a culture that prioritizes the interests of society.

Accordingly, we set the following three values: (1) respect for human life and dignity, (2) responsibility and skills, and (3) act with honesty and integrity. With regard to the second value, SOC-B states that biostatisticians have responsibility and skills and that they must acquire expertise and carry out their work within their competence. Furthermore, the third item establishes the need to work in a way that enables biostatisticians to ensure the appropriateness of their work, accuracy of results, and validity of the science, to develop appropriate relationships with employers and clients so as to avoid pressure or undue influence from others, and to maintain a proper environment.

In the Preamble, the purpose of SOC-B is described as follows: to articulate the mission and value of statisticians and to make the public aware of the roles and responsibilities of statisticians as professionals. It also emphasizes that statisticians and pharmaceutical companies should prioritize the interests of the public, not of the organization, and that the environment should be conducive to the activities of statisticians. In the Background section, the reason for and process of development of SOC-B are provided, as well as the reason for adopting aspirational ethics.

### Standards of Conduct for Stem Cell Researchers

Regarding practices involving stem cell research, historical data and previous experiences suggest that the public has a negative impression towards destroying an embryo and excessive expectations for regenerative medicine. On the other hand, some opined that the guidelines for human ES cell research developed by ministries are either too strict or not in line with current trends, and thus highly likely to hinder research (Nakatsuji, 2007).

Discussions with researchers revealed the need for them to clearly state their stance on the proper handling of blastocysts and somatic cells. The importance of providing adequate explanations and having appropriate relationships with the media and politicians was also pointed out, given the possibility that the public and politicians may misunderstand the ethical issues of research using ES cells and blastocysts, and because the public’s expectations for regenerative medicine are obstacles to sound research. As for ethical issues, it was pointed out that researchers should take the initiative, distinguish between conceptual and practical issues, and avoid reflecting an individuals’ beliefs based on the preferences and feelings in the guidelines of the academic society and ministries.

Based on these perspectives, the following three main values were set: (1) respect human cells, life, and dignity, and the surrounding environment, (2) respect cultural and social values, and conduct activities that respect the ideals/philosophies of regenerative medicine, and (3) embody responsibility and skills, and behave with integrity.

With regard to the first value, SOC-SCR mentions that SCRs should handle blastocysts and somatic cells carefully as treasures of society and refrain from activities that would endanger their value, such as those which would go against public order or those that deviate largely from common sense. The second value reflects Japanese views that have been upheld since ancient times, such as living in harmony with nature.

In addition, the first item of the SOC-SCR mentions the need to exhibit professionalism, that new challenges which have not been experienced in the past require careful consideration, working groups consisting of experts should discuss and decide on new measures and policies, and strive to ensure that contents of the discussions and decisions are reflected in public guideline drafting and revisions by the government and other groups. To address concerns raised by researchers about excessive expectations of the public towards regenerative medicine, the seventh item of the SOC-SCR states that the information will be conveyed in a way that does not mislead the public or patients as to what is being done as research, what is not, and what is clear at this point in time and what can be clarified in the future.

In the Preamble, the purpose of SOC-SCR is described as follows: to clearly state the mission and value of SCRs, and to examine what kind of research and medical treatment can be conducted using stem cells as a professional group, and how a certain technology would be applied. The Background section explains the reason for and process of SOC-SCR development, the reason for adopting aspiration ethics, and the need to have roles and responsibilities as a professional.

## Characteristics of Aspirational SOC

The purpose of our proposal for SOC was to have researchers develop and practice the habit of thinking for themselves what the appropriate action is, and formulate personal standards. Therefore, SOCs are focused not only on presenting universal principles, but also on explaining why researchers should think, and how having personal standards would benefit them as well as others.

In drafting Standards, we avoided expressions such as “do” and “don’t” and instead presented them as a declaration (e.g., “We act in such a way that…”), as in the form of “aspirational ethics” codes. A code in the form of “dos” and “don’ts” can be taken as a command issued from someone outside, which people might obey if it is built in but not without the risk of depriving of their own thinking, vigor, and passion (Deci & Flaste, 1999).

Moreover, since aspirational ethics are not unusual and can be found in various ethical codes such as the Hippocratic Oath, we adopted the aspirational style given its usefulness in reflecting the attitude of professional bodies.

As the underpinning of autonomous activities carried out by professional bodies are expected to play a role in fixing the way research governance is practiced today. In Japan, research regulations such as those concerning clinical trials and application are commonly put in place by first organizing a national council or working group of relevant ministries to formulate national ethical guidelines, and then urging researchers to comply. However, given that the essence of research is to create new knowledge by challenging the unknown, and because risks and benefits of research can be grasped by experts involved in the research themselves, research governance must be maintained autonomously by each professional body (Jones, 2007; Uzawa, 2000). Therefore, the ideal scenario is for the professional bodies to take the initiative to also develop national ethical guidelines regarding their own research field. This, however, has not been achieved likely due to a lack of awareness within the Japanese research community regarding the importance of its own autonomy. By formulating SOC that also serve as a vehicle for declaring the intentions of a professional body, we expected that research communities might be urged to deepen the awareness of their guiding principles. In addition, this aspirational style might help citizens recognize the autonomous stance of the professional body and that they share the same values, which would then promote a more trusting attitude toward researchers (Nakayachi & Cvetkovich, 2008).

Although it is unclear how many researchers and/or groups recognize the importance of aspirational SOC, organizations that follow prohibitive ethical codes are recommended to adopt aspirational SOC when given an opportunity to revise them. In addition, SOC will be beneficial for researcher/workers in areas for which a professional body is difficult to form, or people who work without belonging to an organization, as they can help to collectively raise awareness among researcher/workers.

## Characteristics of “Self-formulated” SOC

As stated in the Preamble, SOCs are a set of shared values and universal principles, and researchers are expected to formulate their personal standards based on SOC. To this end, they need to internalize universal principles, but in doing so, it is important not to introject the principles as they are; rather, the principles need to be digested, accepted, and integrated as one’s own. Once integrated, researchers will then be able to choose the course of their own actions while being aware of universal principles, determine that the source of ideas and actions is consistent with their own values, and act accordingly (Deci & Flaste, 1999). We developed “self-formulated” style SOC for two reasons, as discussed below.

First, it is more difficult for people to deviate from personal (“self-formulated”) standards. In Japan, where the notion of an absolute being such as a monotheistic god is nearly absent, the normative consciousness is low for most people (Yamamoto, 2007). Japanese organizations tend to have vague organizational goals, and rules (if any) within the organization serve as convenient tools for the inner circle in many cases. Moreover, as many are family-type organizations, they tend to be swayed more by feelings than logic (Yamamoto, 2007). Against this cultural backdrop, if the management of a company or research organization highly prioritized profits and performance goals, then injustice might prevail at an organizational level, with the last stronghold being the will of constituent members to choose to do good. If SOC only presented “built-in” standards (dos/don’ts) created by others, then biostatisticians/SCRs might stop thinking for themselves, and more negative effects would be imposed over positive effects (Gohara, 2011). Deviating from the standards would also become easier, as they would find ways to excuse themselves. On the other hand, when one internalizes “self-formulated” standards, to make excuses for oneself would be akin to a self-betrayal; thus, responsible actions can be anticipated.

Second, the formulation of personal standards will provide an opportunity to think about and awaken/cultivate “statistician/researcher-ship” among researchers, workers, and students, many of whom have never contemplated the roles and responsibilities of biostatisticians/SCRs or their missions and values to uphold (Mitcham, 2014; Snieder & Zhu, 2020). In Japan, there is a need to inspire people to think for themselves, as Japanese education is not designed to cultivate the ability to express one’s own opinion (Kariya, 2019) and lacks systematic ethics education, outside the engineering field. Even if people understand the importance of “acting with honesty,” when faced with a dilemma, it is up to them to determine what an honest action is. Based on this understanding, they begin and continue to consider what they want to be, and whom and what they work for, i.e., thinking becomes a habit. At the same time, this will become their inner energy and motivation to “do good research and good work.” Rather than a passive motive to engage in no misconduct, this will instead become a source of motivation and pride to inspire themselves (Iseda, 2008; Kohlberg & Higgins, 1987). Internalizing and incorporating into their own thinking the standards that serve as a source of behavior and attitudes will allow them to become autonomous if they have a clear sense of values and a sense of being guided from within to be in harmony with the universal principles. Only with a personal value system and moral compass would they become capable of enduring and supporting, even in the midst of change and difficulties, their own life with a sense of peace and happiness (Ryff & Keyes, 1995).

Although we expect the readers of SOC to be able to consider “self-formulated” standards based on their imagination and conscience, the task of internalizing universal principles would require an opportunity to think about the ways in which they themselves would behave if faced with a dilemma.

## Practical Example: A Seminar to Encourage Biostatisticians to Consider “Self-formulated” Standards

Provided with the opportunity to teach a class in the graduate school training curriculum, we developed a seminar for biostatisticians to formulate personally meaningful standards based on SOC-B (Table 3). The seminar consisted of a 2.5-h class including lectures and group work, a case study session to think about the meaning of doing responsible work, and a group discussion using a self-development sheet to consider the work biostatisticians do. In the case study, students were asked to consider what they would do if they were biostatisticians in a pharmaceutical company in bad financial shape. In the presented scenario, when they imputed missing data according to the pre-specified statistical analysis plan, they had statistically significant, but clinically inconsistent results, and their superior pressured to submit such inconsistent results to the regulatory agency. After the class, students reflected on the seminar and filled out a self-thinking worksheet, which consisted of questions asking what they want to be and what they need to do as a professional biostatistician. The worksheet was returned to each student with comments from the instructor.

**Table 3:** Outline and contents of the seminar to foster professionalism for biostatisticians

Time (min)	Outline	Contents
5	Orientation	Students are asked to read SOC-B before the seminar The instructor explains that the purpose of the seminar is to formulate personal standards
20	Lecture 1	The instructor explains what professionalism is and why biostatisticians need to create their personal standards by internalizing universal principles
40	Groupwork 1 Case study: i. Role-play ii. Discussion & summarizing	Students consider and discuss how they would act based on a story of biostatistician A who works for a pharmaceutical company B The story is as follows: Pharmaceutical company B is in bad financial shape and needs to increase its profits by marketing a new drug The outcome obtained from the clinical trial of a new drug C has missing data. When A imputed missing data according to the pre-specified statistical analysis plan, A had statistically significant, but clinically inconsistent results. A thinks that such inconsistent results should not be submitted to the regulatory agency, but A’s superior pressures A to submit them, and A is troubled
20	Lecture 2	The instructor explains the contents and background of SOC-B and the necessity to have one’s own standards based on SOC-B for judging what is appropriate for themselves Students are asked to formulate personal standards based on SOC-B
40	Group work 2 Discussion using a self-development sheet & summarizing	Students are asked to consider and discuss what professional biostatisticians do and whom and what they work for based on discussions in the case study The self-development sheet has the following questions: • What value is important to you as a biostatistician? • What are your responsibilities to your colleagues, employers, patients, and the public? • What are barriers to good work? What do you need to do to overcome them?
15	Lecture 3	The instructor asks students to embrace professionalism, and have someone who asks them what they should do in their mind to keep thinking
10	Students’ seminar evaluation	Students write and submit a questionnaire
	Homework A self-thinking worksheet	Students are asked to reflect on the seminar and think about what they want to be and what they need to do as a professional biostatistician and write these down The self-thinking worksheet has the following questions: • What do you think your family, society, and nature have entrusted to you (request or expectation)? • What is the basis for deciding whether you do or don’t do something? • What do you think of the statement, “Companies need to make a profit, and since you are employed, your first priority is to work for the benefit of the company?” • What kind of person do you think you should be as a “professional biostatistician?”

In the lecture, the instructor explained that biostatisticians should be professional and, in order to act responsibly, they need to think and judge for themselves what to do when they encounter a conflict. The instructor asked students to have a basis for judgment and to formulate personal standards by internalizing the universal principles set out in SOC-B.

This seminar was implemented as a part of a graduate course for biostatisticians in a few universities and companies. Our goal is for the seminar to inspire students to examine their own thoughts regarding for whom and what they work for as a professional, and that by continuing to think about their values and standards, they would act responsibly and accomplish good work.

We plan to also develop a seminar for SCRs. Science and technology will continue to develop rapidly, making research more complex and highly specialized. “Self-formulated” standards will be beneficial for organizations such as academic institutions, companies, and research groups in fields where human rights violations and ethical issues may occur out of sight. In particular, in areas such as data science and artificial intelligence, it is difficult for others to verify the legitimacy of work (O'Neil, 2018). Thus, researchers and workers must be prepared to determine for themselves what an appropriate action is.

## Conclusions

We developed SOC-B and SOC-SCR with the aim to urge every biostatistician and SCR to formulate standards. At the same time, we expect professional bodies to address issues and manage quality in an autonomous fashion. We considered self-formulated standards to be necessary, as an appropriate decision cannot be made unless a foundation has been established for considering how to act when confronted with dilemmas or issues. Also, honest behavior is not to be enforced by someone but should come from within the individual based on “who I am and what I work for.”

Our SOCs offer a framework to encourage individuals to formulate their personal standards by thinking about their relationship with society, organizations, and colleagues. If they can view their responsibilities through their own value system, it will help cultivate professionalism. The guidelines of international societies and codes of conduct for scientists will be helpful for professionals to understand basic ethical standards. However, in order to ensure sound practice in each area, we believe it necessary for individuals to internalize universal principles and have the conscience to judge for themselves what an appropriate action is in each situation.

When developing SOCs, researchers and bioethicists should discuss together what values and principles are important and reflect them in SOC, taking into account the characteristics and challenges of research, historical background, and social context. In addition, given the influence of the environment on how humans think, cultural characteristics that are unconsciously shared by different groups should be taken into consideration.

We hope that our SOCs will help inspire and support responsible behavior and integrity, make a positive contribution to sound practice, improve the work environment, cultivate the next generation of professionals, and acquire the trust of society.
